# *Leptospira* Survey in Wild Boar (*Sus scrofa*) Hunted in Tuscany, Central Italy

**DOI:** 10.3390/pathogens9050377

**Published:** 2020-05-14

**Authors:** Giovanni Cilia, Fabrizio Bertelloni, Marta Angelini, Domenico Cerri, Filippo Fratini

**Affiliations:** Department of Veterinary Sciences, University of Pisa, Viale delle Piagge 2, 56124 Pisa, Italy; giovanni.cilia@vet.unipi.it (G.C.); m.angelini5@studenti.unipi.it (M.A.); domenico.cerri@unipi.it (D.C.); filippo.fratini@unipi.it (F.F.)

**Keywords:** leptospirosis, zoonosis, infectious disease, multilocus sequence typing (MLST), wildlife, *Leptospira fainei*, MAT, intermediate *Leptospira*

## Abstract

Leptospirosis is a re-emerging, worldwide zoonosis, and wild boar (*Sus scrofa*) are involved in its epidemiology as the reservoir. The aim of this study was to investigate the prevalence of *Leptospira* with serological, bacteriological, and molecular assays in wild boar hunted in Tuscany (Italy) during two hunting seasons. In total, 287 specimens of sera, kidneys, and liver were collected to perform microscopic agglutination tests (MATs), isolation, and RealTime PCR to detect pathogenic (*lipL32* gene), intermediate (*16S rRNA* gene), and saprophytic (*23S rRNA* gene) *Leptospira*. Within sera, 39 (13.59%) were positive to the MAT, and Australis was the most represented serogroup (4.88%), followed by Pomona (4.18%), and Tarassovi (3.14%). Moreover, four *Leptospira* cultures were positive, and once isolates were identified, one was identified as *L. borgpetersenii* serovar Tarassovi, and three as *L. interrogans* serovar Bratislava. Pathogenic *Leptospira* DNA were detected in 32 wild boar kidneys (11.15%). The characterization through the amplification of the *rrs2* gene highlighted their belonging to *L. interrogans* (23 kidneys), *L. borgpetersenii* (four), and *L. kirschneri* (one), while nine kidneys (3.14%) were positive for intermediate *Leptospira*, all belonging to *L. fainei.* The results of this study confirmed the importance of wild boar in the epidemiology of leptospirosis among wildlife in Central Italy.

## 1. Introduction

Wild boar (*Sus scrofa*) is a large ungulate mammal with worldwide distribution. It can live in several types of habitat, including urban and suburban areas [1,2]. Due to their high adaptability, wild boar populations have rapidly increased in number during recent years, in Europe, and especially in Italy [1,3]. In Italy, wild boar is largely spread in all areas, from the Alps to the southern part of the Italian peninsula, including the islands. There is a high density, particularly in specific regions, such as Tuscany [3,4,5]. The abundant presence of wild boar in the Tuscany region, as well in Central Italy, is suggested by the very high number of animals hunted in this area; every year the hunting of about 42,000 specimens is registered [1,3,4,5]. The massive presence of wild boar in particular areas, other than representing an important source of damage for agriculture [6], can be a severe risk to human and animal health, due to the identification of wild boar as reservoir for many etiological agents; among them typical zoonoses, such as *Leptospira* [7,8,9].

Leptospirosis is a re-emerging zoonotic disease with worldwide spread. It is caused by *Leptospira* spp., a Gram-negative spirochetal bacterium [10,11,12]. The genus *Leptospira* is divided into more than 260 antigenically-different serovars, classified as pathogenic, intermediate, and saprophytic, with different levels of pathogenicity for animals and humans [13,14]. While pathogenic *Leptospira* cause mild or severe infection, intermediate *Leptospira* could possibly be pathogenic, causing mild infection, while saprophytic *Leptospira* are present in the environment and are non-pathogenic [13,14]. Intermediate and saprophytic *Leptospira* could be important due to the strictly-contact and recombination events with pathogenic serovars [15,16,17]. Leptospirosis occurs in tropical, subtropical, and temperate zones, where it is maintained by a large variety of both wild and domestic mammals which can play the role of *Leptospira* maintenance host [18,19,20,21]. The reservoir organisms generally do not develop symptoms, except after a long time [11,12].

*Leptospira* renal-carrying/-colonization/-localization in asymptomatic animals contributes to the maintenance of infection in a particular environment by constantly shedding bacteria through their urine. Accidental contact with *Leptospira-*infected animal urine causes incidental infection, and produces clinical diseases in so-called “incidental hosts” [11,21]. 

Swine, including wild boar and pig, are recognized as maintenance hosts for Pomona, Tarassovi, and Bratislava serovars [21], but can be infected by other *Leptospira* serovars, in relation to both geographic area where the population lives and their behavior [22,23,24,25,26]. The epidemiology of leptospirosis may change over time in domestic and wild animals, and some serovars seems to be prevalent and emerging [26,27]. Moreover, intermediate *Leptospira* DNA has been detected in the kidneys of wild boar hunted in Liguria region (Italy), suggesting a possible infection [7]. 

Tuscany, as well as all of Central Italy, is a geographic area that promotes the presence and the persistence of *Leptospira* in the ecosystem. The features of *Leptospira-*spreading are the presence of several wild animals involved as reservoir, domestic animals bred in extensive farms in contact with wildlife, high presence of hunting activity, and abundance of wetlands, such as marshes, ponds, and irrigation canals [9,26,28,29,30,31,32,33].

The aim of this investigation was to detect and characterize pathogenic, intermediate, and saprophytic *Leptospira* in wild boar hunted in Tuscany region during two hunting seasons (2018/ 2019 and 2019/2020), in order to delineate the risk for the transmission and spreading of leptospirosis to domestic animals and humans.

## 2. Results

**Figure 1:**
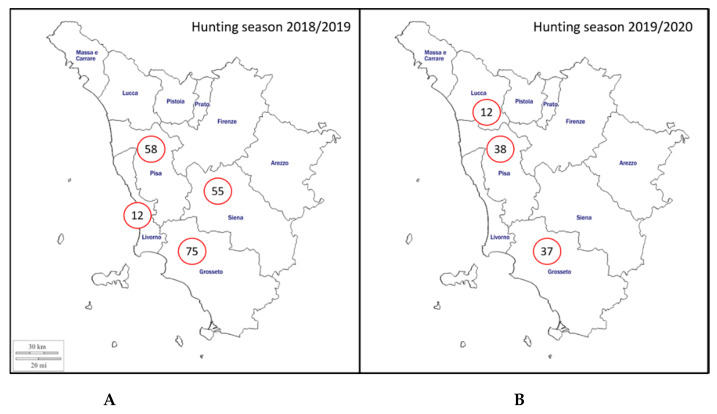
Geographical distribution of the sampling area included in the study (Tuscany region, Italy). The number of sampled hunted wild boar per province is indicated in relation to hunting seasons. (**A**) Hunting season 2018/2019; (**B**) Hunting season 2019/2020.

Serum, kidney, and liver samples were collected from a total of 287 hunted wild boar. Two hundred wild boar were sampled during 2018/2019 hunting season— 75 from Grosseto province, 58 from Pisa province, 55 from Siena province, and 12 from Livorno province (Figure 1). In addition, 87 specimens were sampled during 2019/2020 hunting seasons with 38, 37, and 12 from Pisa, Grosseto, and Lucca provinces, respectively (Figure 1).

Results on distribution of positive sera and kidney for pathogenic *Leptospira* in relation to hunting season, province, sex, and age class are reported in Table 1.

### 2.1. Microscopic Agglutination Test (MAT)

Overall, 39 out of 287 sera (13.59%) were positive in the MAT (Table 2). Considering each hunting season, seropositivity of 16% (32 out of 200 sera) was recorded during 2018/2019, while 8.05% (7 out of 87) was recorded during 2019/2020. Considering wild boar sex, 16 out of 118 male sera (13.55%) and 23 out of 169 (13.61%) were positive in the MAT. Moreover, in relation to age class, 20 out of 142 adult specimens’ sera (14.08%), 7 out of 42 subadult specimens’ sera (16.67%) and 12 out of 100 young specimens’ sera (12.00%) were positive in serological analysis.

Australis resulted the most-recorded serogroup (4.88%), followed by Pomona (4.18%), and Tarassovi (3.14%). Other antibody titers were reported for serogroup Canicola (0.70%) and for serogroups Icterohaemorrhagiae and Ballum (0.45%). The highest titer detected was 1:12,800 for serogroup Tarassovi, followed by titer of 1:6400, which was reported for serogroups Tarassovi and Ballum. 

Results on distribution of positive sera detected by MAT in relation to hunting season, province, sex, and age class are reported in Table 1. No statistical differences (*p* > 0.05) were reported for the serological positivity considering hunting seasons, provinces, and wild boar sex and age class. Moreover, comparing all parameters, no statistical differences (*p* > 0.05) were showed in Pisa and Grosseto during the two different hunting seasons.

### 2.2. Molecular Analysis

Concerning pathogenic *Leptospira,* DNA was detected in 11.15% (32 out of 297) of wild boar kidneys. Table 1 shows PCR-positive kidneys in relation to hunting seasons, province and wild boar sex and age class. During the 2018/2019 and 2019/2020 hunting seasons, 15.5% (31 out of 200) and 1.15% (1 out of 87) of PCR positivity was reported among kidneys samples, respectively. Considering wild boar sex, 12 out of 118 male sera (10.16%) and 20 out of 169 (11.83%) scored positive. Moreover, in relation to age class, 10 out of 142 adult specimens’ kidneys (7.04%), 6 out of 42 subadult specimens’ kidneys (14.28%), and 16 out of 100 young specimens’ kidneys (16.00%) gave positive results in serological analysis.

No statistical differences (*p* > 0.05) were highlighted comparing province, wild boar sex, or age class. Conversely, the incidence of pathogenic *Leptospira-*positive kidneys was statically higher (*p* ≤ 0.01) during 2018/2019 hunting season compared to the 2019/2020 ones. 

The detection of pathogenic *Leptospira* DNA was higher (*p* ≤ 0.01) during 2018/2019 hunting season in both Pisa and Grosseto provinces compared to the second hunting season. On the contrary, there were no statistical differences (*p* > 0.05) in the Pisa and Grosseto provinces during the two different hunting seasons, comparing sex and age class of wild boar. 

The 3.14% (9 out of 287) of kidneys were positive for intermediate *Leptospira*. The positivity in relation to hunting seasons, province, wild boar sex, and age class are showed in Table 1. All the intermediate *Leptospira-*positive kidneys (4.5%; 9 out of 200) were collected in 2018/2019, highlighting a statistical difference (*p* ≤ 0.01) in relation to 2019/2020 hunting season. Also, the results showed a statistically-higher infection rate (*p* ≤ 0.01) in male compared to female, and in Pisa province compared to other provinces. No statistical difference (*p* > 0.05) were noted among age classes. 

No saprophytic *Leptospira* DNA was detected in kidney samples. No positive reactions were recorded in wild boar livers across all specimens during the two year of investigation.

### 2.3. Leptospira spp. Isolation, Characterization and Genotyping 

Four *Leptospira* cultures were positive after 30 days of incubation. The results, reported in Table 3, show that three isolates were obtained from subadult males hunted in Pisa province, while the other one was from an adult female hunted in Livorno. Through multilocus sequence typing (MLST) analysis, one isolate was identified as *Leptospira borgpetersenii* serogroup Tarassovi serovar Tarassovi (Sequence Type 153), while the other three were identified as *L. interrogans* serogroup Bratislava serovar Bratislava (ST 24), as reported in Table 3. Moreover, the amplification of the *rrs2* gene from kidney tissue highlights that the species belonged to *L. borgpetersenii* and *L. interrogans*, respectively.

**Figure 2:**
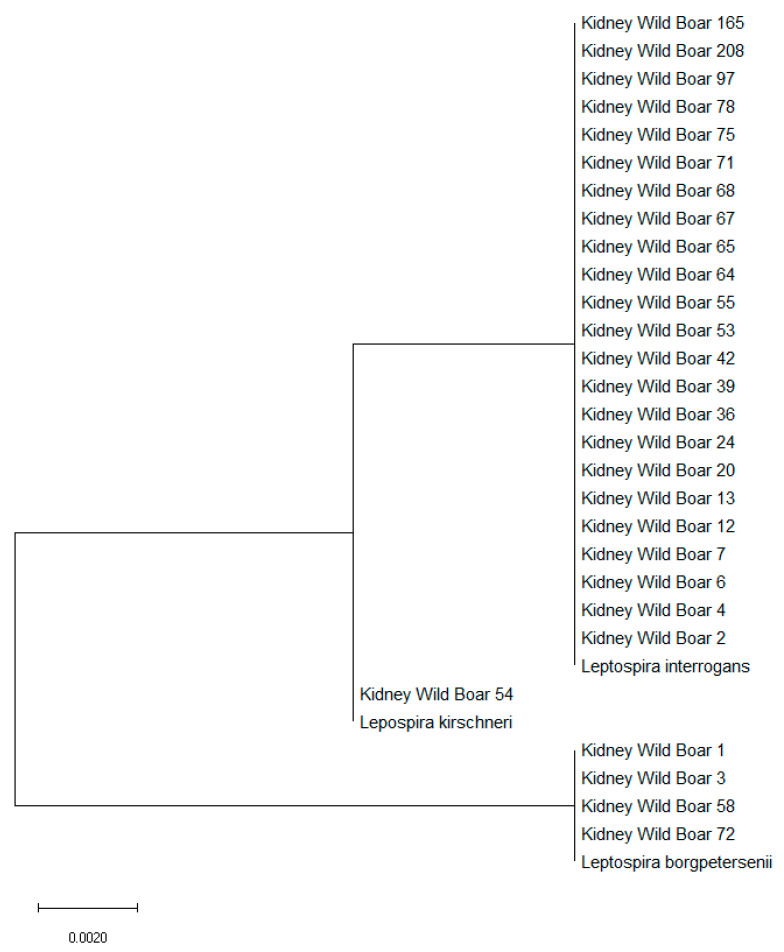
Molecular phylogenetic analysis for the *rrs2* gene of *Leptospira interrogans, Leptospira borgpetersenii,* and *Leptospira kirschneri* by the maximum likelihood method, based on the Tamura–Nei model. The branch lengths of the tree measured the number of substitutions per site. The analysis involved 31 nucleotide sequences. There was a total of 452 positions in the final dataset.

With regard to characterization of PCR-positive samples, amplification of the *rr2* gene highlighted that pathogenic *Leptospira* belonged to *L. interrogans* (23 kidneys), *L. borgpetersenii* (four) and *L. kirschneri* (one) (Table 4). Moreover, phylogenetic analysis identified the close relationship to their respective *Leptospira* species. (Figure 2).

**Figure 3:**
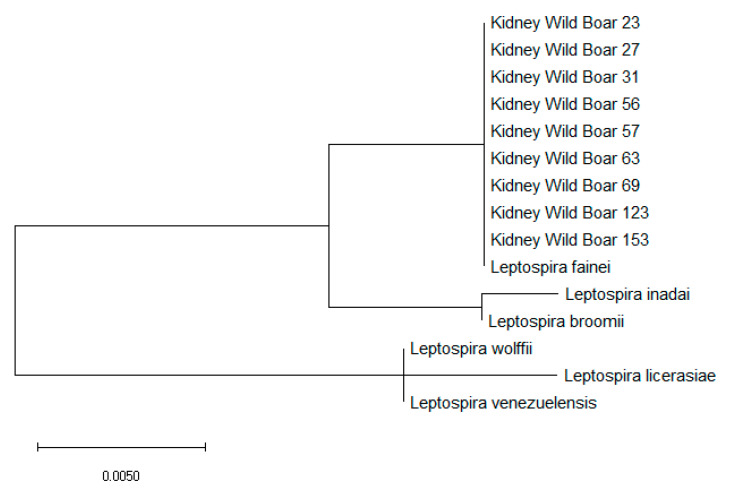
Molecular phylogenetic analysis for 16s rRNA gene of *Leptospira fainei*, *Leptospira inadai*, *Leptospira broomii*, *Leptospira wolffii*, *Leptospira licerasiae*, and *Leptospira venezuelensis* by the maximum likelihood method based on the Tamura–Nei model. The branch lengths of the tree measured the number of substitutions per site. The analysis involved 22 nucleotide sequences. There was a total of 438 positions in the final dataset.

Moreover, the amplification of intermediate *Leptospira 16s rRNA* DNA of PCR-positive specimens showed *L. fainei in* all nine kidneys (Table 5). Furthermore, the phylogenetic analysis identified the close relationship to *L. fainei* specie. (Figure 3).

## 3. Discussion

Leptospirosis is a re-emerging worldwide public health risk, but is underestimated and characterized by a downward trend [34]. Climatic changes, rainfall, modifications of ecological niches, and new potential maintenance hosts all represent important features involved in *Leptospira* epidemiology.

Wild boar, among wildlife, is an important *Leptospira* reservoir and, for several areas, represents an appropriate indicator for this zoonotic infectious disease.

In this investigation we reported the results of serological analysis, isolation and molecular investigations performed on 287 hunted wild boar during two hunting seasons (2018/2019 and 2019/2020).

With regard to serological assay, the prevalence of *Leptospira* infection, recorded in both hunting seasons, was very similar to other studies carried out on wild boar in Tuscany [9,22,26,31,33]. Moreover, the seroprevalence reported in this investigation was very close to other data obtained in different Italian regions [27,35,36,37,38]. Unfortunately, serological data about leptospirosis in wildlife, especially regarding wild boar, are available just in some regions. It also seems that *Leptospira* serovars/serogroups have a different geographical distribution, suggesting a distinct circulation/epidemiology in other environments/ecosystems. Examining each region, Australis, Pomona, and Tarassovi, are the most-detected serogroups in the Tuscany region [22,26,31,33], In the Lombardy and Emilia Romagna regions it is Bratislava [37,38,39], in the Campania region it is Tarassovi [35], whereas in the Sardinia region it is Pomona and Grippotyphosa [36]. 

The distribution of *Leptospira* serovars in wild boar in Europe is also not homogeneous; high levels of Pomona infection was recorded in Germany, Croatia, Poland, and Spain [23,40,41,42]. Bratislava was the most-detected serovar in Sweden [25], Tarassovi in Portugal and Slovenia [24,43], Grippotyphosa in Czech Republic [44], and Hardjo in Poland [23].

Little information is available on *Leptospira* isolation in wild boar, especially in Italy [38]. The obtained *Leptospira* isolates, identified by MLST, confirm the circulation of Tarassovi and Bratislava serogroups within wild boar in Tuscany. Bratislava isolation is commonly performed in wildlife due to the high spectrum of maintenance hosts [45,46,47], while Tarassovi is rarely isolated and detected through serology. Indeed, Tarassovi is strictly a swine-specific serovar; its isolation, reported in this investigation, seems to confirm the hypothesis that wild boar could serve as reservoir of Tarassovi [26,43,48]. Only two of them (Bratislava, isolated from subadult from Pisa province; Table 3) reported correlated serological positivity for serogroup Australis at titer 1:100, while the other two gave negative results in the MAT. No correlation was found between the MAT and PCR-positive results. The seronegativity of *Leptospira*-positive kidneys has been previously reported for other animal species [49,50,51,52], including swine [47], suggesting an early or chronic infection.

Conversely to serological results, very few studies were performed on pathogenic *Leptospira* DNA in wild boar kidneys. In spite of this, the prevalence of pathogenic *Leptospira* infection reported during the years of this investigation was very close to the results obtained in Northern Italy (11.02%) [38] and in the Liguria region (12.13%) [7]. Moreover, prevalences of 10.30% and 15.3% were found in two different investigations performed in Japan [53,54], while 3.40% was reported in the USA [55]. Based on phylogenetic analysis, pathogenic *Leptospira* DNA in wild boar kidney belong to *L. interrogans, L. borgpetersenii*, and *L. kirschneri.* With respect to the serovars in Italy that are more often detected through isolation or serology, [27,38,56,57,58,59] and the other serovars that are rarely seropositive [28,60], it might be hypothesized that *L. kirschneri* species found in wild boar kidneys could be related to serogroup Grippotyphosa, while *L. borgpetersenii* species could be related to the serogroups Tarassovi or Ballum. On the other hand, it is very difficult to infer the serogroup related to *L. interrogans* positivity, due to the inclusion of Icterohaemorrhagiae, Canicola, Pomona, Australis, and Sejroe serogroups in this species. Probably, in relation to serological and bacteriological results obtained in this study, the identified *L. interrogans* could belong to serogroup Australis.

The data reported in this investigation suggest that the liver does not seem be a *Leptospira* target organ in wild boar. Furthermore, it could exclude an early stage of infection (leptospiremia) and confirm that positive animals are only chronic renal carriers, as also suggested by isolation from kidneys. 

If very little information is available on pathogenic *Leptospira* DNA in wild boar, there is even less data on intermediate *Leptospira*. To the best of these authors’ knowledge, it was only in the Liguria region of Italy that 0.49% of wild boar kidneys were positive for intermediate *Leptospira* DNA in the same year of this investigation [7]. As Liguria and Tuscany are two adjoining Italian regions, a large wild boar movement could be a feature of these regions [61,62,63]. Even though the species of intermediate *Leptospira* from Liguria were not identified, those found in this investigation belong to *L. fainei* species. *L. fainei* was isolated for the first time from fig and was detected in human sera in Australia [64,65] and a human infection with febrile status was reported in France (from a Portuguese citizen) and in two patients in Denmark [66,67]. Considering wild boar behavior and its ability to live in anthropomorphic environment, a transmission between human and wildlife could be possible. As these are the first determination in European wildlife, more studies are needed to understand the epidemiology of this intermediate *Leptospira* that could causes severe infection in humans [65,66,67].

The statistical difference presented during the hunting seasons between pathogenic and intermediate *Leptospira* incidence in wild boar could be related to the temperature and the amount of rainfall recorded in Tuscany during these periods. As reported in literature, rainfall and temperature influence the incidence of leptospirosis in humans and animals [12,68,69,70,71,72,73,74,75]. Indeed, from 2018 to 2019, the temperatures and the rainfall were both higher than those from 2019 to 2020 [76,77,78,79,80,81], suggesting that these atmospheric phenomena could be involved in these seasonality incidence differences.

## 4. Materials and Methods 

### 4.1. Sample Collection

During two hunting seasons (the first from November 2018 to January 2019 and the second from November 2019 to January 2020) hunted wild boar blood, kidney, and liver were sampled. Blood samples were collected by ocular puncture [82]. The boar’s age class was determined after assessing the degree of tooth eruption and the wear and tear of teeth of the lower jaw, considering three age classes: young (under 12 months old), sub-adult (between 12 and 24 months), and adult (over 24 months old). The animal’ sex was also recorded [83].

All animals were hunted in the Tuscany region during authorized hunting seasons (November–January), following the regional hunting law (Regolamento di attuazione della legge regionale 12 gennaio 1994, n. 3 D.P.G.R. 48/R/2017). No animals were specifically sacrificed for this study purpose. Animals did not present gross lesions related to infectious disease at postmortem examination, performed during sampling operations.

### 4.2. Microscopic Agglutination Test (MAT)

Blood samples were centrifugated at 10,000 rpm for 10 minutes to obtain the serum. In order to detect *Leptospira* antibodies, sera were tested through microscopic agglutination test (MAT) [84]. Titer of 1:100 was considered as positive. For the MAT, live *Leptospira* antigens used were: *Leptospira interrogans* serovar Icterohaemorrhagiae (serogroup Icterohaemorrhagiae, strain RGA), *L. interrogans* serovar Canicola (serogroup Canicola, strain Alarik), *L. interrogans* serovar Pomona (serogroup Pomona, strain Mezzano), *L. kirschneri* serovar Grippotyphosa (serogroup Grippotyphosa, strain Moskva V), *L. borgpetersenii* serovar Tarassovi (serogroup Tarassovi, strain Mitis Johnson), *L. interrogans* serovar Bratislava (serogroup Australis, strain Riccio 2), *L. interrogans* serovar Hardjo (serogroup Sejroe, serovar Hardjoprajitno), and *L. borgpetersenii* serovar Ballum (serogroup Ballum, strain Mus 127).

### 4.3. Leptospira spp. Isolation 

Each wild boar organ was cultured in Ellinghausen-McCullough-Johnson-Harris (EMJH) medium (Difco, Detroit, MI, USA). Approximately 10 cm³ from each organ was homogenized with 5 mL of sterile water and 1 mL of homogenate was cultured in 5 mL of EMJH. Cultures were incubated at 30 °C ± 1 °C for 120 days and observed every 10 days under dark-field microscopy to evaluate possible bacterial growth.

### 4.4. Molecular Analysis

From each kidney and liver, DNA was extracted using Quick-DNA Plus Kits (Zymo Research, Irvine, CA, USA) following the manufacturer’s instructions. 

Two different multiplex Realtime-PCR were employed; the first, targeting *Leptospira* spp. (*16S rRNA* gene) and pathogenic *Leptospira* (*lipL32* gene), was performed on all samples [85,86]. The second protocol was only performed on positive *Leptospira* spp. and negative *lipL32* samples, targeting intermediate *Leptospira* (*16S rRNA* gene) and saprophytic *Leptospira* (*23S rRNA* gene) [16,86]. As a positive control for the *lipL32* gene, DNA extracted from a pure culture of *Leptospira interrogans* serogroup Pomona strain Mezzano was used. As a positive control for the 23S rRNA gene for saprophytic *Leptospira*, DNA extracted from a pure culture of *Leptospira biflexa* serogroup Patoc strain Patoc I was used. As a negative control, sterilized saline water was used. A total reaction volume of 15 μL was prepared by using 2x QuantiTect Probe PCR Master Mix (Qiagen, Hilden, Germany), 2 μM of each primer, 500 nM of each probe, and 3 μL of DNA, as previously reported [7]. The RealTime-PCR assay was performed on a Rotorgene Corbett 6000 (Corbett Research, Sydney, Australia) with the following thermal conditions: a holding stage of 95 °C for 5 min and 45 cycles of 95 °C for 15 s and 60 °C for 30 s. Samples with Ct *lipL32* < 35 were considered positive and those samples with 35 < Ct lipL32 ≥ 40 were repeated.

### 4.5. Leptospira spp. Characterization and Genotyping

First, serogroups of the isolates were determined through the MAT using a panel of eight polyclonal anti-sera against the eight serovars reported in Section 4.2. The agglutination with specific antiserum was used to identify the presumptive strain’s serogroup [84].

Isolated *Leptospira* were genotyped using a multilocus sequence typing (MLST) scheme based on housekeeping genes [87,88,89].

Moreover, the *Leptospira* species were identified from positive pathogenic and intermediate *Leptospira* PCR reactions, using primer for *rrs2* gene and *16S rRNA* gene, respectively [86,88].

The amplification of each target gene was realized with HotStarTaq Master Mix Kit (Qiagen, Hilden, Germany), and further sequenced (BMR Genomics, Padova, Italy) using the same amplification primer sets and analyzed using BioEdit Software [90]. Phylogenetic analysis was performed by the maximum likelihood method based on the Tamura–Nei model using MEGA 10 software [91].

### 4.6. Statistical Analysis

Data were analyzed with chi-square (X^2^) test. The statistical test was used to evaluate the *Leptospira* infection ratio in relationship to sex (male or female), age class (young, sub-adult, or adult), province (Pisa, Lucca, Livorno, Grosseto, or Siena) and hunting season (2018/2019 or 2019/2020). Statistical significance threshold was set at a p value ≤ 0.05 [92].

## 5. Conclusions

In conclusion, this investigation confirms through the MAT, isolation, and molecular assays, the role of wild boar in the epidemiology of leptospirosis in Central Italy. Wild boar represents a good indicator of *Leptospira* circulating in a specific area where many different animal species share the same environment. Furthermore, wild boar populations are able to live in a wide spectrum of habitat types, and, have recently reached sub-urban and urban areas. In Italy, little recent data on human leptospirosis are available; however, some studies investigated the prevalence of infection in risk categories (hunters, farmers, and forestry workers) showing serological positivity to *Leptospira* [93,94]. Moreover, on the basis of the most recent report on human leptospirosis in Italy [95], a high infection rate was recorded in adult males, and this could indicate that leptospirosis is strictly related to worker activity. Hunters, for example, are usually all male and over 30 years old. In particular, these peoples are exposed to an high risk of infection due to management and slaughtering of dead animals being performed with little health care [96].

Tarassovi and Bratislava are the two main serogroups that circulate within wild boar in Tuscany. Although Bratislava has been more detected, the isolation of Tarassovi suggests that wild boar could be the main reservoir. In addition, as for pathogenic *Leptospira*, the presence of intermediate species in wild boar kidney underlines the need to perform other studies aimed at understanding the newly- emerging species, *L. fainei*, in animals and in humans.

## Figures and Tables

**Table 1:** Distribution of positive sera and kidney for pathogenic *Leptospira* in relation to hunting season, province, sex, and age class.

Hunting Season	Province	Sex	Age Class	Examined Wild Boar	MAT-Positive Sera (%)	PCR-Positive Kidneys (%)
2018/2019	Pisa	Male	Adult	9	2 (22.2)	1 (11.1)
(n = 200)	(n = 58)	(n = 30)	Subadult	10	2 (20.0)	4 (40.0)
			Young	11	3 (27.3)	0
		Female	Adult	14	2 (14.3)	2 (14.3)
		(n = 28)	Subadult	5	1 (20.0)	2 (40.0)
			Young	9	1 (11.1)	2 (22.2)
	Grosseto	Male	Adult	10	2 (20.0)	2 (20.0)
	(n = 75)	(n = 29)	Subadult	5	1 (20.0)	0
			Young	14	1 (7.1)	3 (21.4)
		Female	Adult	22	2 (9.09)	1 (4.6)
		(n = 46)	Subadult	5	0	0
			Young	19	2 (10.5)	4 (21.1)
	Siena	Male	Adult	10	2 (20.0)	0
	(n = 55)	(n = 22)	Subadult	4	1 (25.0)	0
			Young	8	0	1 (12.5)
		Female	Adult	21	5 (23.8)	3 (14.3)
		(n = 33)	Subadult	2	0	0
			Young	10	1 (10.0)	3 (30.0)
	Livorno	Male	Adult	2	0	0
	(n = 12)	(n = 4)	Subadult	0	0	0
			Young	2	1 (50.0)	1 (50.0)
		Female	Adult	4	1 (25.0)	1 (25.0)
		(n=8)	Subadult	0	0	0
			Young	4	2 (50.0)	1 (25.0)
2019/2020	Pisa	Male	Adult	6	0	0
(n = 87)	(n = 38)	(n = 13)	Subadult	4	0	0
			Young	3	0	0
		Female	Adult	21	2 (9.52)	0
		(n = 25)	Subadult	1	1 (100)	0
			Young	3	0	1 (33.3)
	Grosseto	Male	Adult	11	1 (9.09)	0
	(n = 37)	(n = 16)	Subadult	1	0	0
			Young	4	0	0
		Female	Adult	10	1 (10.0)	0
		(n = 21)	Subadult	5	1 (20.0)	0
			Young	6	1 (16.7)	0
	Lucca	Male	Adult	1	0	0
	(n=12)	(n = 4)	Subadult	0	0	0
			Young	3	0	0
		Female	Adult	4	0	0
		(n = 8)	Subadult	0	0	0
			Young	4	0	0

**Table 2:** Numbers of positive serological reactions detected for wild boar sera in relation to different *Leptospira* serogroups at low (1:100) and high titers (1:12,800).

*Leptospira* Serogroup	Titer								Total (%)
	100	200	400	800	1600	3200	6400	12800	
Icterohaemorrhagiae			1						1 (2.56%)
Canicola		1	1						2 (5.13%)
Pomona	8	1	3						12 (30.8%)
Grippotyphosa									
Tarassovi	4	1			1	1	1	1	9 (23.1%)
Australis	5		5	2	1	1			14 (35.9%)
Sejroe									
Ballum							1		1 (2.56%)
Total	17	3	10	2	2	2	2	1	39 (100%)

**Table 3:** Characterization of wild boar *Leptospira* isolates tested with anti-sera and multilocus sequence typing (MLST).

Sample	Wild Boar			Isolates Characterization	
	Sex	Age Class	Province	Anti-Serum MAT Serogroup	MLST (Sequence Type)
Kidney 5	Male	Subadult	Pisa	Tarassovi	Tarassovi (ST 153)
Kidney 14	Male	Subadult	Pisa	Australis	Bratislava (ST 24)
Kidney 15	Male	Subadult	Pisa	Australis	Bratislava (ST 24)
Kidney 22	Female	Adult	Livorno	Australis	Bratislava (ST 24)

**Table 4:** Characterization of *Leptospira* species in wild boar pathogenic *Leptospira-*positive PCR-amplifying *rrs2* gene.

Sample	Wild Boar			Isolate Characterization
	Sex	Age Class	Province	*Leptospira S*pecies
Kidney 1	Female	Young	Pisa	*L. borgpetersenii*
Kidney 2	Female	Subadult	Pisa	*L. interrogans*
Kidney 3	Male	Adult	Pisa	*L. borgpetersenii*
Kidney 4	Female	Young	Pisa	*L. interrogans*
Kidney 6	Male	Young	Siena	*L. interrogans*
Kidney 7	Female	Young	Siena	*L. interrogans*
Kidney 12	Female	Young	Siena	*L. interrogans*
Kidney13	Female	Adult	Siena	*L. interrogans*
Kidney 20	Male	Young	Grosseto	*L. interrogans*
Kidney 24	Female	Young	Livorno	*L. interrogans*
Kidney 36	Female	Adult	Grosseto	*L. interrogans*
Kidney39	Female	Young	Grosseto	*L. interrogans*
Kidney 42	Female	Young	Siena	*L. interrogans*
Kidney 53	Female	Young	Grosseto	*L. interrogans*
Kidney 54	Male	Young	Grosseto	*L. kirschneri*
Kidney 55	Male	Young	Grosseto	*L. interrogans*
Kidney 58	Female	Adult	Pisa	*L. borgpetersenii*
Kidney 64	Female	Adult	Siena	*L. interrogans*
Kidney 65	Female	Adult	Siena	*L. interrogans*
Kidney 67	Female	Adult	Pisa	*L. interrogans*
Kidney 68	Female	Subadult	Pisa	*L. interrogans*
Kidney 71	Male	Adult	Grosseto	*L. interrogans*
Kidney 72	Female	Young	Grosseto	*L. borgpetersenii*
Kidney 75	Male	Subadult	Pisa	*L. interrogans*
Kidney 78	Male	Young	Livorno	*L. interrogans*
Kidney 97	Male	Adult	Grosseto	*L. interrogans*
Kidney 165	Female	Young	Grosseto	*L. interrogans*
Kidney 208	Female	Young	Pisa	*L. interrogans*

**Table 5:** Characterization of *Leptospira* species in wild boar intermediate *Leptospira-*positive PCR-amplifying *16s rRNA* gene.

Sample	Wild Boar			Isolate Characterization
	Sex	Age Class	Province	*Leptospira S*pecies
Kidney 23	Male	Young	Livorno	*L. fainei*
Kidney 27	Male	Adult	Pisa	*L. fainei*
Kidney 31	Female	Adult	Pisa	*L. fainei*
Kidney 56	Male	Young	Grosseto	*L. fainei*
Kidney 57	Male	Adult	Pisa	*L. fainei*
Kidney 63	Male	Adult	Siena	*L. fainei*
Kidney 69	Female	Subadult	Pisa	*L. fainei*
Kidney123	Male	Adult	Livorno	*L. fainei*
Kidney 153	Male	Adult	Siena	*L. fainei*
